# Preparation and luminescent properties of color-adjustable La_1-x-y_SiBO_5_: xTb^3+^, yEu^3+^ phosphor for NUV-LEDs

**DOI:** 10.1371/journal.pone.0344199

**Published:** 2026-03-13

**Authors:** Wenyu Zhao, Jiacheng Zhao, Lele Gao, Huihui Cao, Xuening Ding, Songbo Li

**Affiliations:** 1 School of Chemistry and Chemical Engineering, Inner Mongolia University of Science and Technology, Baotou, China; 2 Rare Earth Advanced Materials Technology Innovation Center, Inner Mongolia Northern Rare Earth Advanced Materials Technology Innovation Co., Ltd., Baotou, China; Purdue University, UNITED STATES OF AMERICA

## Abstract

A series of La_1-x-y_SiBO_5_:xTb^3+^, yEu^3+^ phosphors was synthesized through high-temperature solid-state reaction. The crystal structure, morphology, and luminescence properties of the phosphors were characterized by employing X-ray diffraction, scanning electron microscopy, photoluminescence, photoluminescence excitation and fluorescence lifetime measurements. Results show that the excitation spectra of La_1-x_SiBO_5_:xTb^3+^ and La_1-y_SiBO_5_:yEu^3+^ overlap at 300–400 nm, and the La_1-x-y_SiBO_5_:xTb^3+^, yEu^3+^ phosphors emit yellowish green (530–560 nm) and red (580–650 nm) light under 377 nm excitation. When x = 0.05 and y increases from 0.005 to 0.05, the emission intensity of Tb^3+^ decreases gradually, whereas that of Eu^3+^ increases gradually. This trend indicates energy transfer from Tb^3+^ to Eu^3+^ and color change from green to yellow and then red. The fluorescence lifetime test further confirms energy transfer from Tb^3+^ to Eu^3+^. The prepared LaSiBO_5_:Tb^3+^, Eu^3+^ phosphors exhibit effective tunable emission and near ultraviolet excitation and thus have potential applications in White-LEDs.

## 1. Introduction

Phosphor-converted white light emitting diodes (PC-WLEDs), which are composed of phosphors and blue/NUV semiconductor chips, have considerable applications in solid-state lighting because of their excellent color stability and luminescence performance [1,2]. As an important component of PC-WLED devices, phosphors have a remarkable influence on device application performance, such as color rendering index (CRI), correlation color temperature (CCT), luminescence efficiency, and thermal stability. Among factors, the choice of phosphors is the most important for good CRI and stability, especially for full-spectrum LEDs that are similar to the solar spectrum with high CRI and considered as healthy lighting for human beings. Blue, green, and red phosphors pumped by near-ultraviolet chips can achieve full-spectrum LEDs with higher CRI and lower CCT compared with conventional WLEDs composed of blue chips and yellow YAG:Ce^3+^ phosphors. Given that phosphors of different matrices have different degrees of thermal stability and water resistance, a color shift occurs during phosphor service. Hence, doping the same phosphor matrix with different luminous centers produces a wide band of colors and similar reliability over time. Research has proven that Tb^3+^→Eu^3+^ energy transfer is effectively color tunable. Examples include Mg_2_SiO_4_:Tb^3+^, Eu3+ [3], Ca_8_ZnM (PO_4_)_7_ (M = Lu/Tb, Lu/Eu, and Tb/Eu) [4], and Sr_3_Y(BO_3_)_3_:Tb^3+^, Eu3+ [5]. All reports have confirmed that energy transfer between Tb^3+^ and Eu^3+^ occurs through dipole–dipole interaction. For the choice of the phosphor matrix, borate-based host materials are excellent luminescent materials that have the advantages of a remarkably low synthesis temperature from borate and good chemical stability from silicate and exhibits potential for application in LED lamps and displays [6–9]. The specific LaSiBO_5_ host matrix of phosphors has not been reported yet. In this study, Tb^3+^- and Eu^3+^-codoped LaSiBO_5_ phosphors with adjustable color were prepared through traditional high-temperature solid-state reaction for the first time, and their luminous properties were studied in detail.

## 2. Materials and methods

### 2.1. Preparation

The raw materials SiO_2_ (A.R.), H_3_BO_3_ (A.R.), La_2_O_3_ (99.99%), Tb_4_O_7_ (99.99%), and Eu_2_O_3_ (99.99%) were weighed in accordance with the stoichiometric ratios of the La_1-x_SiBO_5_:xTb^3+^(x = 0.03–0.15), La_1-y_SiBO_5_:yEu^3+^(y = 0.09–0.25), and La_1-x-y_SiBO_5_:0.05Tb^3+^, yEu^3+^ (y = 0.001, 0.005, 0.01, 0.02, 0.03, 0.05) phosphors. The ingredients were thoroughly ground in an agate mortar, then transferred into a crucible, and sintered at 1150 °C for 5 h in air atmosphere. After cooling to room temperature, the final product was obtained.

### 2.2. Characterization

X-ray diffraction (XRD, D8 ADVANCE, Bruck) was used to determine the crystal structures of the samples. The radiation source was the Cu target *K*_*α*_ (*λ* = 0.15406 nm), and the scanning 2θ range was 10°–80°. The surface morphology of the phosphors was analyzed through scanning electron microscopy (SEM, QUANTA 400, FEI). Photoluminescence–photoluminescence excitation was measured with a fluorescent spectrometer (Hitachi, F-4600) with a 150 W xenon lamp as the excitation source. Luminescence decay curves were recorded with a combined fluorescence lifetime and steady-state spectrometer (Edinburgh FLS920). All the above tests were conducted at room temperature.

## 3. Results and discussion

**Fig 1:**
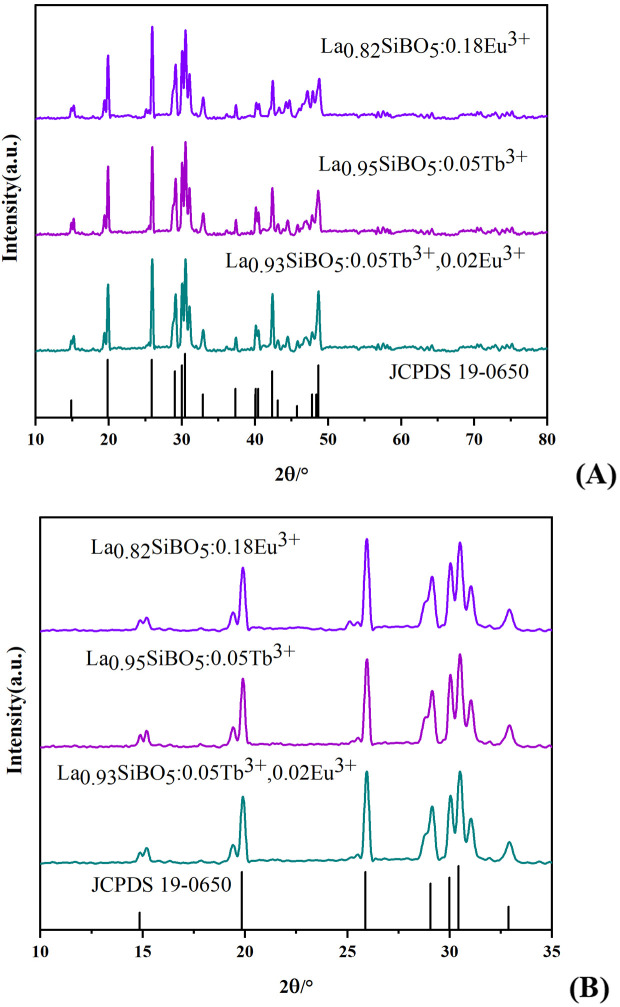
XRD patterns. (A) LaSiBO_5_:Tb^3+^, Eu^3+^ phosphors and JCPDS card. (B) Magnified local image.

The XRD patterns of the La_0.95_SiBO_5_:0.05Tb^3+^, La_0.82_SiBO_5_:0.18Eu^3+^, and La_0.93_SiBO_5_:0.05Tb^3+^, 0.02Eu^3+^ samples are shown in Fig 1(a). LaSiBO_5_ is trigonal, and its crystal structure parameters are a = 6.876 Å, b = 6.876 Å, c = 6.744 Å, and *V* = 281.05 Å^3^. All diffraction peaks of the samples are consistent with those in the JCPDS#19–0650 standard card, indicating that doping with Tb^3+^ and Eu^3+^ does not change the crystal structure of the matrix and does not produce any impurity phases. The XRD diﬀraction peaks of the samples slightly deviate to the higher angle, indicating that the interplanar crystal spacing *d* in the sample is smaller than that in the pure phase LaSiBO_5_. In accordance with the radius and valence similarity principle, when La^3+^ is replaced by Tb^3+^ and Eu^3+^ in the matrix, the ionic radii of Eu^3+^ and Tb^3+^ become slightly smaller than that of La^3+^ (*R*_La_ = 106 pm, *R*_Eu_ = 95 pm, and *R*_Tb_ = 92 pm), resulting in a decrease in *d* value. It will lead to lattice distortion and diﬀraction peak splitting [10]. Fig 1(b) shows the magnified local image which can demonstrate the diffraction peak splitting clearly.

**Fig 2:**
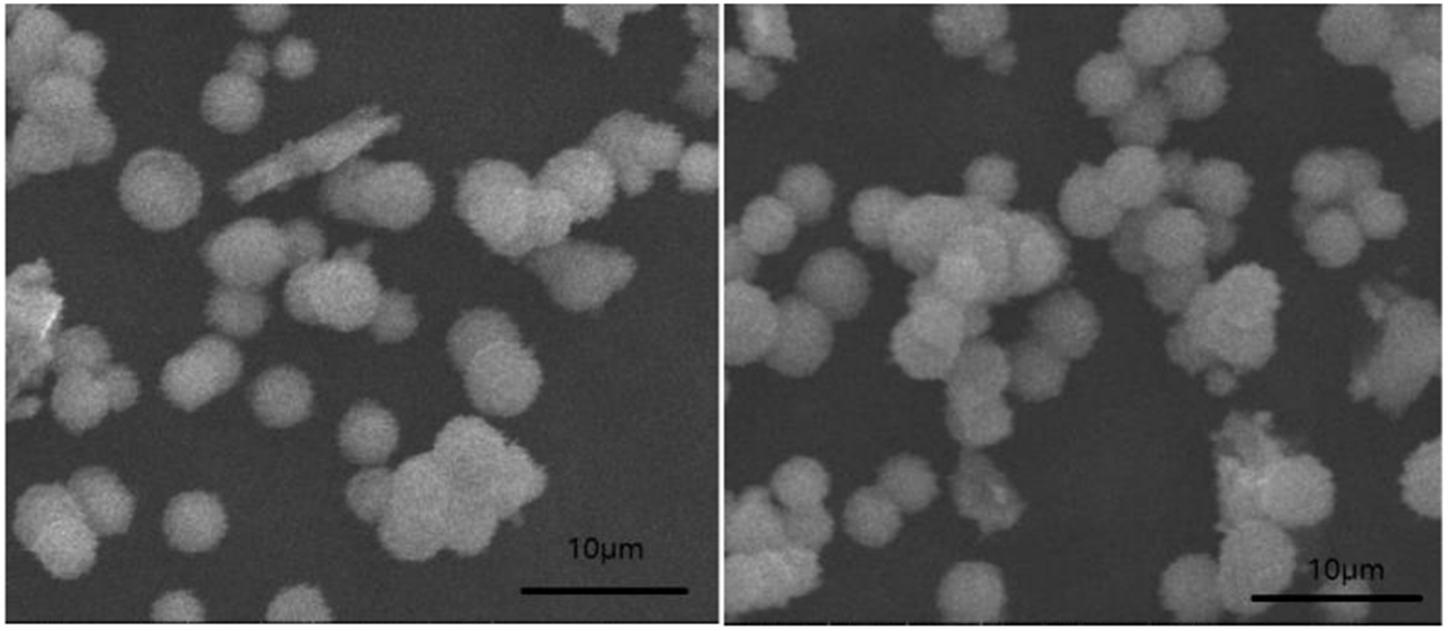
Morphology of La_0.93_SiBO_5_:0.05Tb^3+^, 0.02Eu^3+^ sample.

In terms of the relationship between phosphor morphology and luminous performance, particle size homogeneity is evidently beneficial for facile coating and uniform color. However, ground particles that are too small destroy crystal structure and degrade luminous performance. The particle size of phosphor coatings on chips should be uniform and less than 20 μm. The SEM images of the La_0.93_SiBO_5_:0.05Tb^3+^, 0.02Eu^3+^ sample are shown in Fig 2. All the particles are spherical and uniform in size with slight agglomeration. The particle size is mostly distributed below 5 μm, which is applicable to LED packaging.

**Fig 3:**
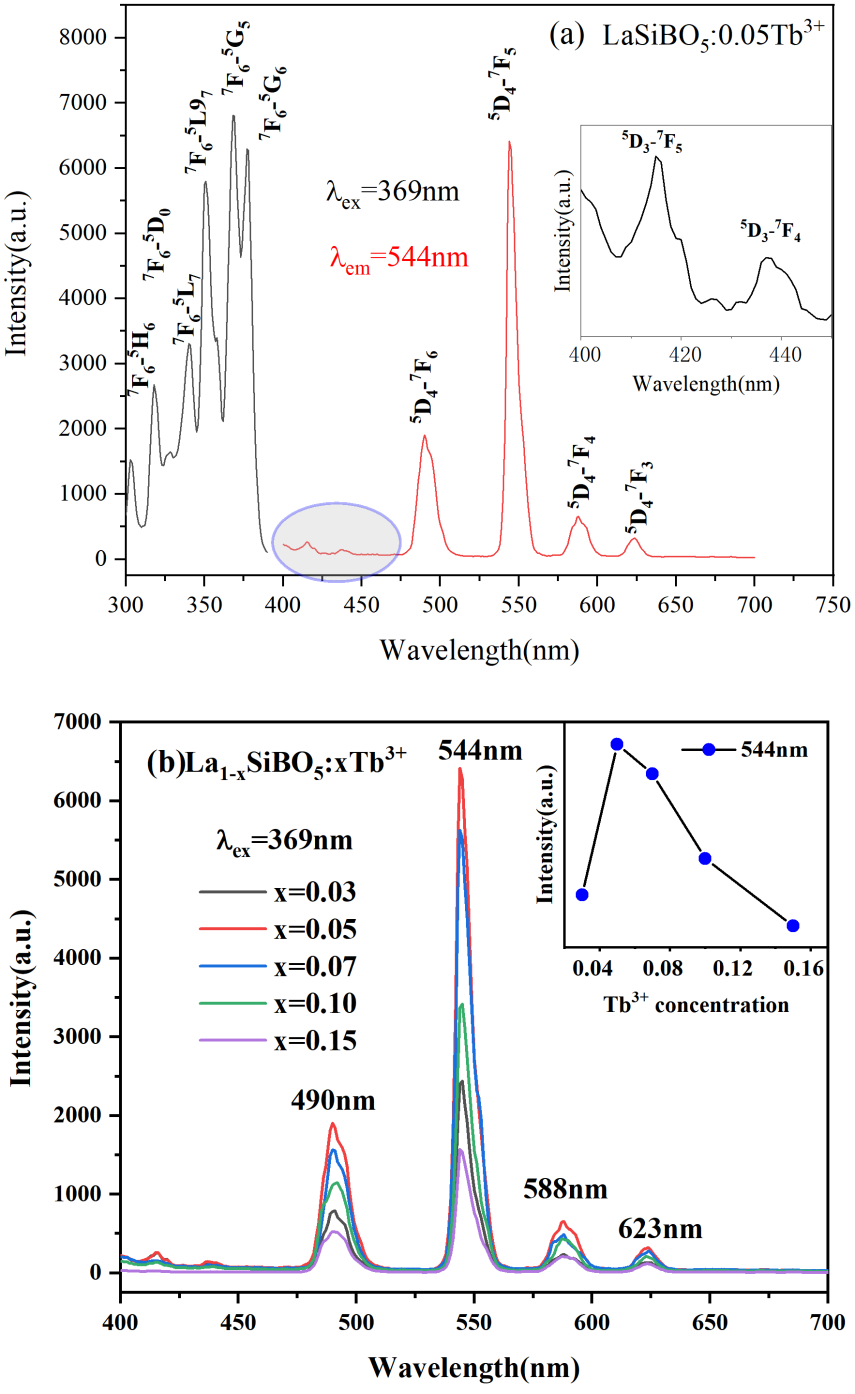
Photoluminescence spectra of LaSiBO_5_:Tb^3+^. (A) Excitation spectra. (B) Emission spectra of La_1-x_SiBO_5_:xTb^3+^ phosphors.


Fig 3(a) shows that when 369 nm NUV light is used as the excitation wavelength, the emission peaks are located at 415, 439, 490, 544, 588, and 623 nm, corresponding to the ^*5*^*D*_*3*_*→*^*7*^*F*_*5*_, ^*5*^*D*_*3*_*→*^*7*^*F*_*4*_, ^*5*^*D*_*4*_*→*^*7*^*F*_*6*_, ^*5*^*D*_*4*_*→*^*7*^*F*_*5*_, ^*5*^*D*_*4*_*→*^*7*^*F*_*4*_, and ^*5*^*D*_*4*_*→*^*7*^*F*_*3*_ energy level transitions of Tb^3+^, respectively [11,12]. Among these energy level transitions, ^*5*^*D*_*4*_*→*^*7*^*F*_*5*_ at 544 nm is the strongest. Under 544 nm monitoring, the excitation peaks are located at 303, 318, 341, 351, 369, and 377 nm, corresponding to the ground state ^7^F_6_ and the excited states ^*5*^*H*_*6*_, ^*5*^*D*_*0*_, ^*5*^*L*_*7*_, ^*5*^*L*_*9*_, ^*5*^*G*_*5*_, and ^*5*^*G*_*6*_ of Tb^3+^ [13,14]. The strongest excitation peak is located at 369 nm. In general, the doping amount of rare earth ions is an important factor for the emission intensity of phosphors. The emission spectra of La_1-x_SiBO_5_:xTb^3+^ were collected under the same test conditions shown in Fig 3(b). The emission spectra show the same shape as the Tb^3+^ doping amount increases. However, the emission intensity gradually increases and reaches the maximum value when the Tb^3+^ concentration is 0.05. When the Tb^3+^ concentration exceeds 0.05, the luminescence intensity begins to decrease. This phenomenon, which is known as concentration quenching, is caused by the migration of excited energy between luminous ions or to quenching centers, leading to a loss of excited energy through nonradiative transitions [15]. The concentration quenching mechanism can be explained in accordance with the Blasse formula below [16]:

$$R_{c}=\text{2}[\frac{\text{3}V}{\text{4}\pi x_{e}Z}{]}^{\text{1}/\text{3}},$$ (1)

where *R*_*C*_ is the critical distance, *V* represents the unit cell volume (281.05 Å^3^), *N* is the number of cations in the unit cell (6), and *x*_*e*_ is the optimal concentration of Tb^3+^ (0.05). The mechanism is exchange interaction when the critical distance is less than 5 Å and is multipolar interaction otherwise. Calculation shows that *R*_*C*_ = 12.14 Å, which is considerably larger than 5 Å. Therefore, the concentration quenching of La_1-*x*_SiBO_5_:*x*Tb is mainly based on multipolar interaction. Three types of multipole interactions exist: dipole–dipole (d–d), dipole–quadrupole (d–q), and quadrupole–quadrupole (q–q). They can be calculated with the formula [17]:


$$\text{lg}\left(\frac{\text{1}}{x}\right)=\text{C}-(\theta /\text{3})\text{lg}x,$$ (2)

where *I* is the luminous intensity; *x* represents the activator content, %; C is a constant; and *θ* is a multipolar interaction function. *θ* values of 6, 8, and 10 represent d–d, d–q, and q–q interactions, respectively. The emission intensity at 544 nm under 369 nm excitation was measured when 0.03 ≤ *x* ≤ 0.15. The linear relationship between lg (*I/x*) and lg (*x*) was linearly fitted by using Origin2021 software, and the slope of the straight line was obtained as −(*θ*/3) = −2.34563, that is, *θ* ≈ 6. As can be inferred from this result, the concentration quenching mechanism of La_1-*x*_SiBO_5_:*x*Tb(0.03 ≤ *x* ≤ 0.15) is the d–d interaction.

**Fig 4:**
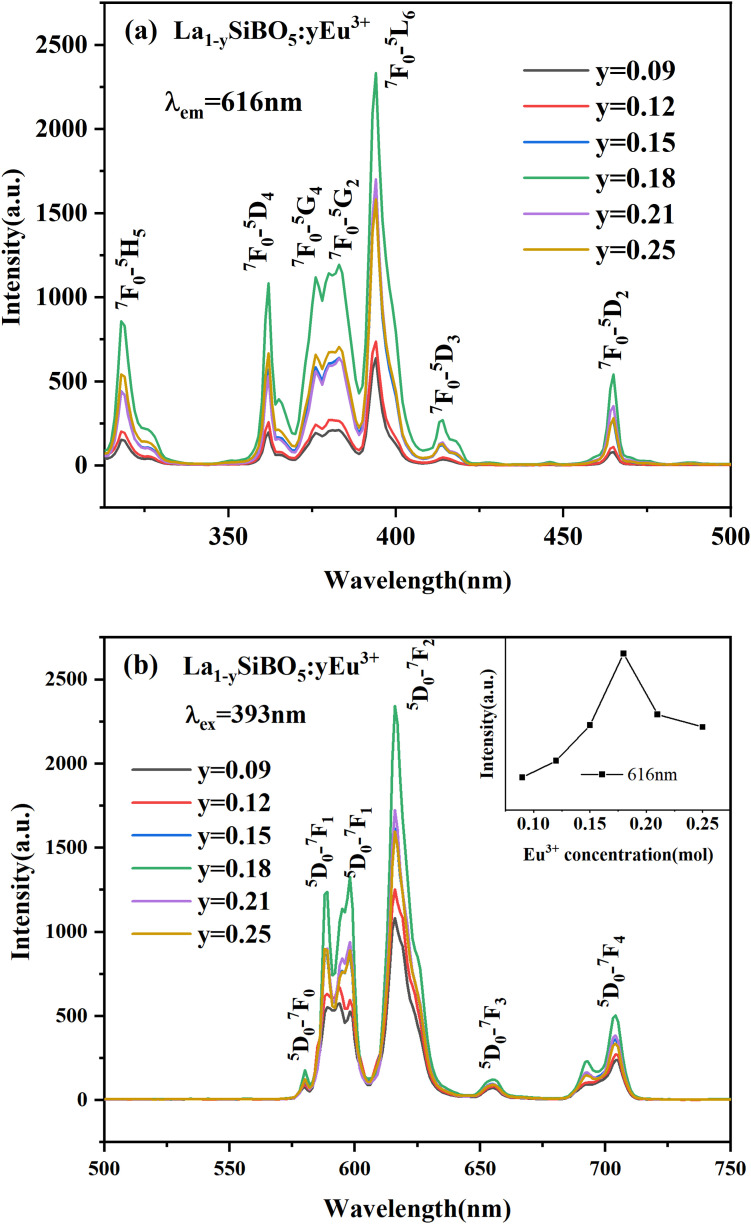
Photoluminescence spectra of LaSiBO_5_:Eu^3+^. (A) Excitation spectra. (B) Emission spectra of La_1-y_SiBO_5_:yEu^3+^ phosphors.

Fig 4(a) shows no significant difference in the shape and position of excitation spectra among the phosphor samples with different doping concentrations. However, a considerable difference in intensity is observed. The excitation spectra of La_1-y_SiBO_5_:yEu^3+^ are composed of strong *4f–4f* electron transition absorption (300–500 nm), and the main excitation peaks are located at 362, 376, 393, and 465 nm. The strongest excitation peaks at 393 nm correspond to the ^*7*^*F*_*0*_*–*^*5*^*L*_*6*_ energy level transition of Eu3+ [18]. Fig 4(b) shows that the main peaks of the emission spectrum are located at 580 and 589 nm, corresponding to the ^*5*^*D*_*0*_*–*^*7*^*F*_*0*_ characteristic transition and Eu^3+^ MD transition, respectively. The main peaks of the emission spectra are located at 598, 616, 654, and 703 nm and are attributable to the ^*5*^*D*_*0*_*–*^*7*^*F*_*1*_, ^*5*^*D*_*0*_*–*^*7*^*F*_*2*_, ^*5*^*D*_*0*_*–*^*7*^*F*_*3*_, and ^*5*^*D*_*0*_*–*^*7*^*F*_*4*_ characteristic transitions of Eu^3+^, respectively [19,20]. In accordance with the energy level distribution characteristics of Eu^3+^, ^*5*^*D*_*0*_*–*^*7*^*F*_*2*_ dominates the spectrum and belongs to the electric dipole transition. The strongest emission peak is located at 616 nm. The symmetry of Eu^3+^ in the lattice can be deduced from the emission characteristics of ^*5*^*D*_*0*_*→*^*7*^*F*_*J*_ (*J* = 0, 1, 2, 3). When Eu^3+^ occupies inversely symmetric positions, its magnetic dipole transition ^*5*^*D*_*0*_*→*^*7*^*F*_*1*_ dominates and emits orange light. Conversely, if Eu^3+^ is located in a noninversely symmetric position, then the red emission of the electric dipole transition ^*5*^*D*_*0*_*→*^*7*^*F*_*2*_ is stronger than that of the magnetic dipole transition ^*5*^*D*_*0*_*→*^*7*^*F*_*1*_. The emission color is affected by the environment of the matrix crystal field and is related to the crystallographic position occupied by Eu3+ [21]. Given that the emission peak corresponding to the ^*5*^*D*_*0*_*→*^*7*^*F*_*2*_ transition has greater intensity than that corresponding to the ^*5*^*D*_*0*_*→*^*7*^*F*_*1*_ transition, the material mainly emits red light.

**Fig 5:**
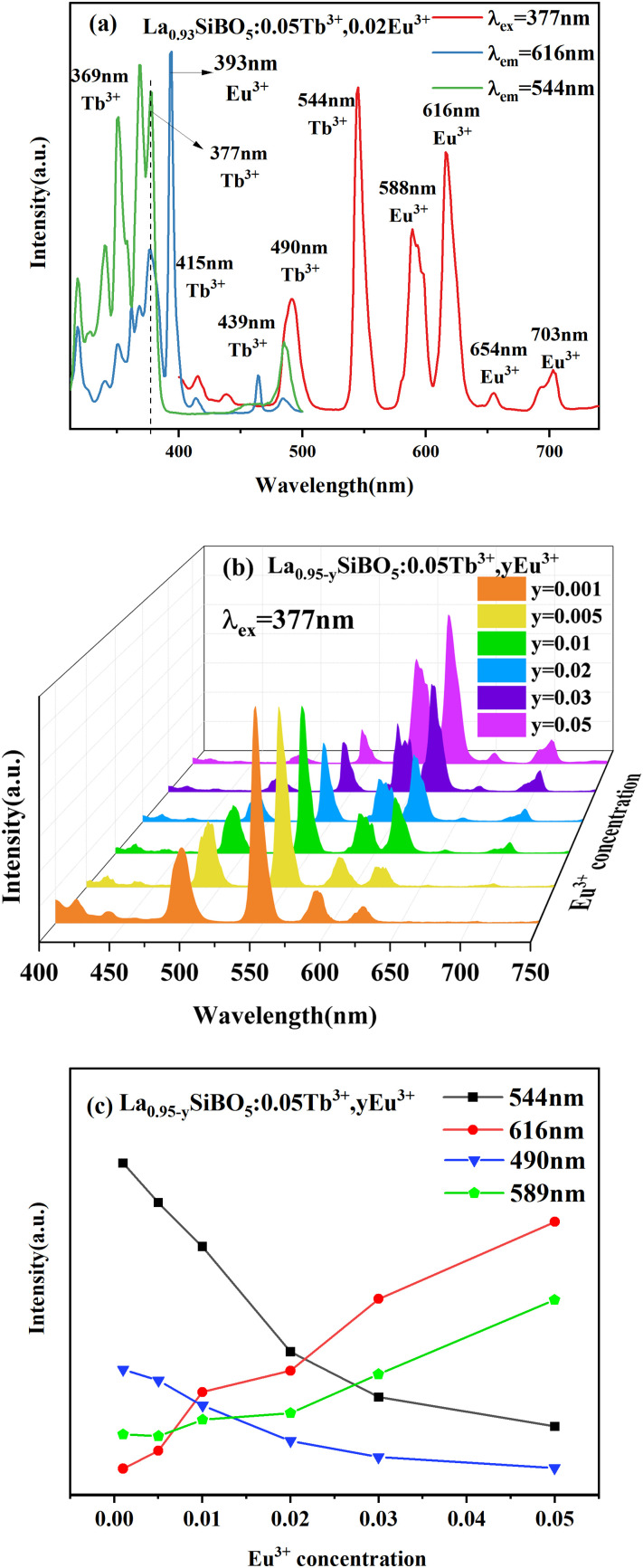
Photoluminescence spectra of LaSiBO_5_:Tb^3+^, Eu^3+^. (**A) Excitation** and emission spectra of **La**_0.93_SiBO_5_:0.05Tb^3+^, 0.02Eu^3+^phosphor. (b) Emission spectra of La_0.95-y_SiBO_5_:0.05Tb^3+^, yEu^3+^ phosphors. (c) Eu^3+^ concentration dependence of emission intensity.

Fig 5(a), (b), and (c) show the excitation and emission spectra of La_0.93_SiBO_5_:0.05Tb^3+^, 0.02Eu^3+^; the excitation and emission spectra of La_0.95-y_SiBO_5_:0.05Tb^3+^, yEu^3+^ samples; and the emission intensities at 490, 544, 589, and 616 nm of La_0.95-y_SiBO_5_:0.05Tb^3+^, yEu^3+^ samples. Fig 5(a) displays that at the excitation wavelength of 377 nm, the fluorescence spectra of La_0.93_SiBO_5_:0.05Tb^3+^, 0.02Eu^3+^ show the typical red emission of Eu^3+^ and the typical green emission of Tb^3+^. The emission spectrum of Tb^3+^ and the excitation spectrum of Eu^3+^ overlap at 400–425 nm, proving the possibility of energy transfer. The emission peaks at 491 and 544 nm observed under 377 nm excitation are attributed to the ^*5*^*D*_*4*_*→*^*7*^*F*_*6*_ and ^*5*^*D*_*4*_*→*^*7*^*F*_*5*_ energy level transitions of Tb^3+^, respectively. In addition, the emission peaks at 590 and 616 nm correspond to the characteristic transitions of ^*5*^*D*_*1*_*→*^*7*^*F*_*1*_ and ^*5*^*D*_*0*_*–*^*7*^*F*_*2*_ from Eu^3+^, respectively. The excitation spectrum of La_0.93_SiBO_5_:0.05Tb^3+^, 0.02Eu^3+^ was monitored at 616 nm, as shown in Fig 5(a). The excitation spectrum shows the typical excitation peaks of Tb^3+^ (377 nm) and Eu^3+^ (393 nm). However, when monitored at the typical Tb^3+^ emission of 544 nm, the excitation spectrum of La_0.93_SiBO_5_:0.05Tb^3+^, 0.02Eu^3+^ does not show the characteristic excitation peaks of Eu^3+^. Therefore, the energy transfer of LaSiBO_5_:Tb^3+^, Eu^3+^ has occurred, and the energy transfer of Tb^3+^→Eu^3+^ is irreversible [22]. A series of La_0.95-y_SiBO_5_:0.05Tb^3+^, yEu^3+^ (y = 0, 0.001, 0.005, 0.01, 0.02, 0.03, 0.05) phosphors was prepared to determine the optimal concentration of Tb^3+^/Eu^3+^ codoping in the LaSiBO_5_ matrix. The excitation and emission spectra of the Tb^3+^- and Eu^3+^-codoped samples are shown in Fig 5(b). All the spectra have similar shapes but not peak height. With the gradual increase in Eu^3+^ concentration, the intensity of the emission peaks at 590 and 616 nm gradually increases and that of the emission peaks at 491 and 544 nm gradually decreases. As shown in Fig 5(c), with the increase in the Eu^3+^ concentration in the La_0.95-y_SiBO_5_:0.05Tb^3+^, yEu^3+^ phosphor, the intensities of emission from Eu^3+^ at 589 and 616 nm increase and those from Tb^3+^ at 490 and 544 nm decrease. Therefore, the color of the L_a1-x-y_SiBO_5_:xTb^3+^, yEu^3+^ phosphors can be effectively regulated by controlling the doping concentration ratio of Eu^3+^:Tb^3+^.

**Fig 6:**
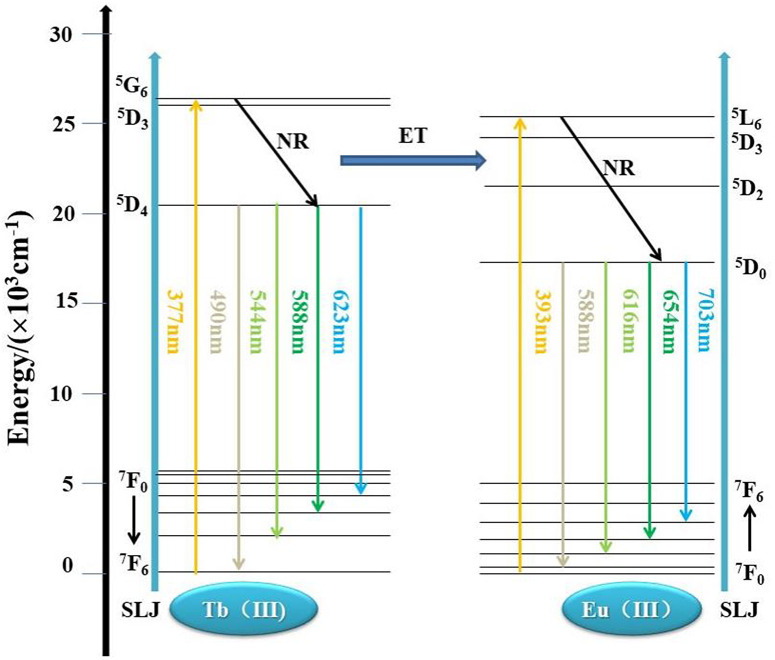
Energy level transitions of Tb^3+^ and Eu^3+^ in La_1-x-y_SiBO_5_: Tb^3+^, Eu^3+^.

Fig 6 shows the energy level transition diagram of LaSiBO_5_:Tb^3+^, Eu^3+^. When the energy level of Tb^3+^ ions transits to high excited states by external excitation, it usually reaches the ^5^D_4_ energy level through nonradiative transition. Some of these electrons return to the ground state (^*5*^*D*_*4*_*→*^*7*^*F*_*6*_, ^*7*^*F*_*5*_, ^*7*^*F*_*4*_, ^*7*^*F*_*3*_), resulting in the characteristic emission of Tb^3+^. Meanwhile, the energy of the Tb^3+^ energy level transition (^*5*^*D*_*4*_*→*^*7*^*F*_*6*_, ^*7*^*F*_*5*_, ^*7*^*F*_*4*_, ^*7*^*F*_*3*_) is likely to be absorbed by the high excitation levels of Eu^3+^ (^*7*^*F*_*0*_*→*^*5*^*D*_*0*_, ^*5*^*D*_*1*_), and red emission is observed through the ^*5*^*D*_*0*_*–*^*7*^*F*_*J*_ (*J* = 1, 2, 3, 4) radiative transition. The emission of Tb^3+^ ions overlaps effectively with the excitation of Eu^3+^. In addition, the Tb^3+^ energy level at ^*5*^*D*_*4*_ does not return to the ground state to produce green light emission but instead relaxes to the ^*5*^*D*_*1*_ energy level and then to the ^*5*^*D*_*0*_ energy level of Eu^3+^ through nonradiative transition. It then transfers to the ^*7*^*F*_*J*_ energy level through radiation transition, generating the red light emission of Eu^3+^. Thereafter, typical Tb^3+^ green emission and Eu^3+^ red emission occur simultaneously [23].

**Fig 7:**
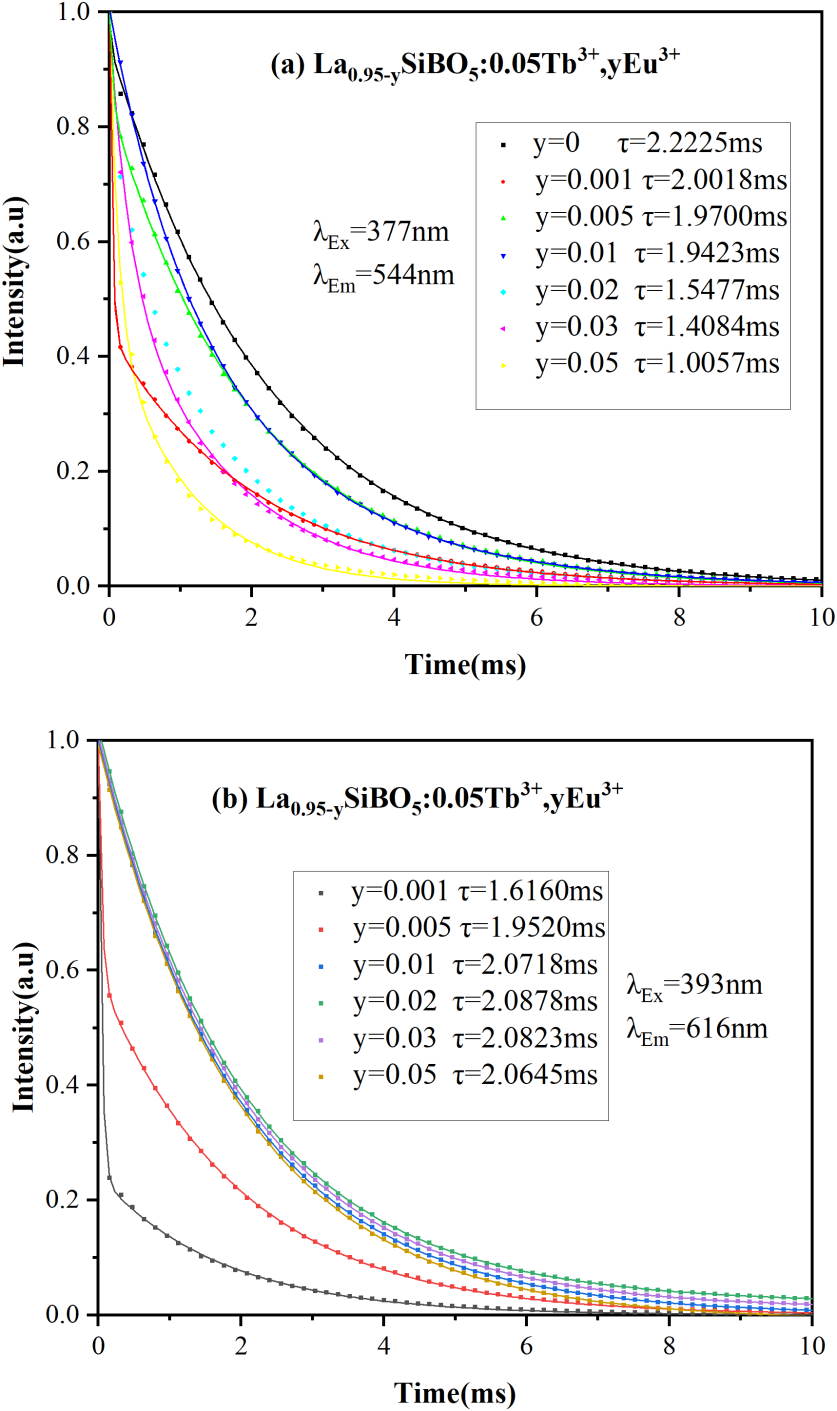
Decay curves of La_0.95-y_SiBO_5_:0.05Tb^3+^, yEu^3+^ phosphors.


The lifetime decay curves of the prepared phosphors are depicted in Fig 7(a) and 7(b) to confirm further the energy transfer between Eu^3+^ and Tb^3+^ in La_0.95-y_SiBO_5_:0.05Tb^3+^, yEu^3+^. The decay curves of the codoped rare earth ions conform to double exponential behavior. Decay lifetime can be calculated by using the following equation [24]:

$$I_{\left(t\right)}=A_{\text{1}}exp(-t/\tau_{\text{1}})+A_{\text{2}}exp(-t/\tau_{\text{2}})$$ (3)

where *I*_*(t)*_ is the luminescence intensity at time *t*; A_1_ and A_2_ are decay constants; and *τ*_*1*_ and *τ*_*2*_ are the rapid and slow lifetimes of the fluorescence lifetime, respectively. The average lifetime (*τ*) can be calculated by using the following equation [25]:


$$\tau *=(A_{\text{1}}\tau_{\text{1}}^{\text{2}}+A_{\text{2}}\tau_{\text{2}}^{\text{2}})/(A_{\text{1}}\tau_{\text{1}}+A_{\text{2}}\tau_{\text{2}})$$ (4)

The decay curve under the excitation of 377 nm and the emission of 544 nm is shown in Fig 7(a). The fluorescence lifetime of the phosphor without Eu^3+^ doping is 2.2225 ms. When Eu^3+^ concentrations increase to 0.001, 0.005, 0.01, 0.02, 0.03, and 0.05, *τ* decreases gradually to 2.0018, 1.9700, 1.9423, 1.5477, 1.4084, and 1.0057 ms, respectively. For further proof of the remarkable energy transfer from Tb^3+^ to Eu^3+^ in LaSiBO_5_:Tb^3+^, Eu^3+^, the energy transfer efficiency (*η*) and energy transfer probability (*P*_*t*_) can be calculated by using the following formulae:


$$H=\text{1}-\tau_{s}/\tau_{so}$$ (5)

$$P_{t}=\text{1}/\tau_{s}-\text{1}/\tau_{so}$$ (6)

where *τ*_*so*_ and *τ*_*s*_ represent the decay lifetime of Tb^3+^ in the absence and presence of Eu^3+^, respectively. *η* and *P*_*t*_ values increase significantly with the increase in Eu^3+^ concentration [26,27]. At the concentrations of 0.001, 0.005, 0.01, 0.02, 0.03, and 0.05, the *η* values of Eu^3+^ are 9.93%, 11.36%, 12.61%, 30.36%, 36.63%, and 54.75%, respectively, and the *P*_*t*_ values are 4.97%, 5.77%, 6.50%, 19.62%, 26.01%, and 54.44%, respectively. Therefore, energy has transferred from Tb^3+^ to Eu^3+^. The decay curves of the La_0.95-y_SiBO_5_:0.05Tb^3+^, yEu^3+^ phosphors at 616 nm emission and 393 nm excitation are shown in Fig 7(b). When the Eu^3+^ doping concentrations are 0.001, 0.005, and 0.01, the corresponding lifetimes increase markedly to 1.6160, 1.9520, and 2.0718 ms, indicating Tb^3+^→Eu^3+^ energy transfer [28]. When the Eu^3+^ doping concentrations are 0.02, 0.03, and 0.05, the corresponding lifetimes are 2.0878, 2.0823, and 2.0645 ms. The decrease in fluorescence lifetime is small because of the slow decrease in luminous intensity from Tb^3+^ that hinders energy transfer. The critical distance (*R*_*C*_) is an important index for analyzing the energy transfer mechanism between luminous centers. The Tb^3+^ → Eu^3+^ energy transfer can occur through exchange interactions or electro–multipolar interactions. *R*_*C*_ can be approximated by employing the Blasse formula (1). *Xc* is the total critical concentration of Tb^3+^ and Eu^3+^. It is a part of the exchange interaction mechanism for *R*_*C*_ < 5 Å and a multipolar interaction mechanism for *R*_*C*_ > 5 Å. The sample La_0.93_SiBO_5_:0.05Tb^3+^, 0.02Eu^3+^ has *V* = 265 Å^3^, *X*_*C*_ = 0.07, and *N* = 6. *R*_*C*_ was calculated as 10.64 Å > 5 Å. The electrical multipole interaction is therefore responsible for energy transfer from Tb^3+^ to Eu^3+^.

**Fig 8:**
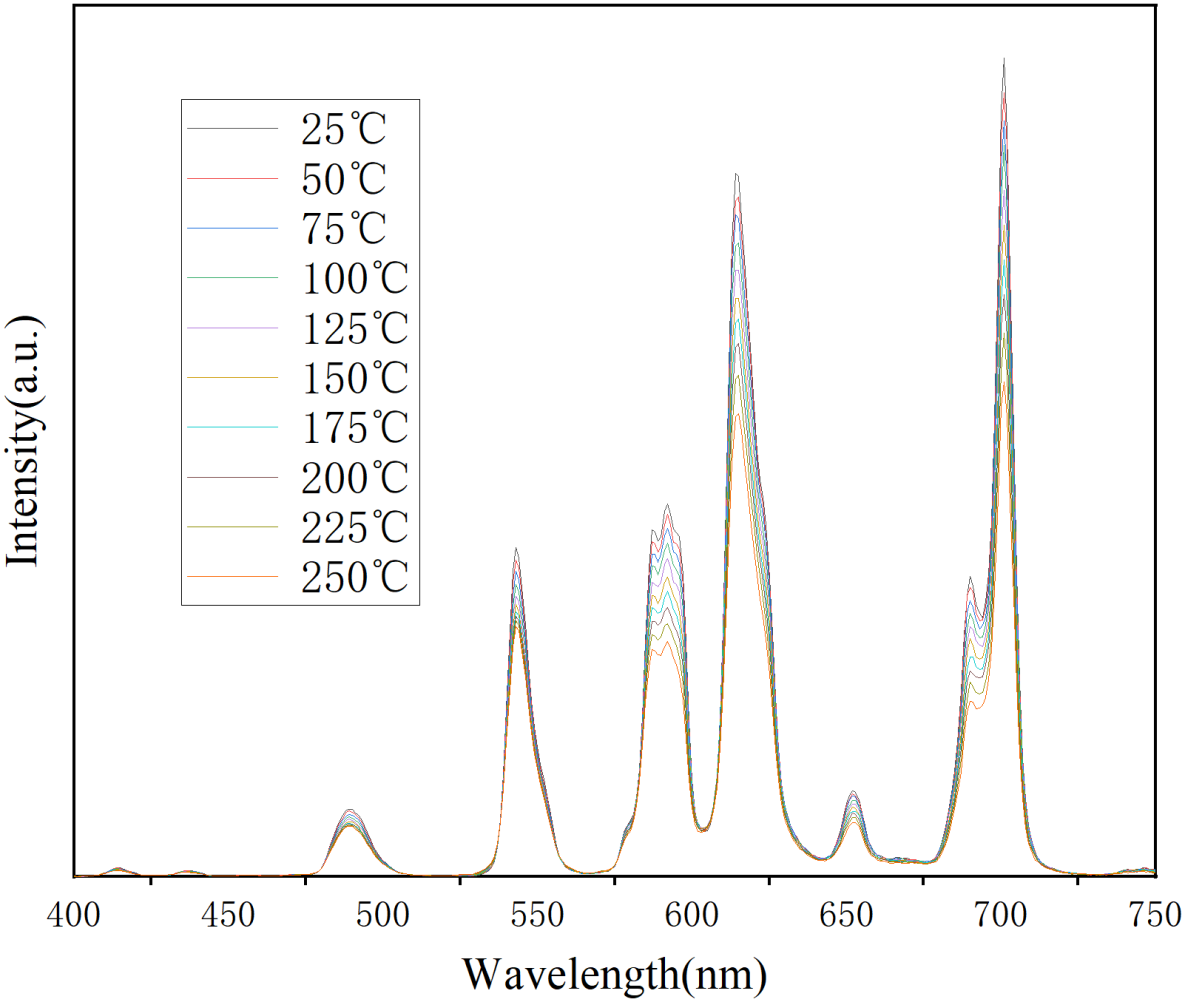
Temperature-dependant emission spectra of La_0.92_SiBO_5_: 0.05Tb^3+^, 0.03Eu^3+^ phosphor.

Fig 8 shows the temperature-dependent emission spectra of the La_0.92_SiBO_5_:0.05Tb^3+^, 0.03Eu^3+^ phosphor. The shape of the emission spectra does not change during heating. The characteristic emission intensities of Tb^3+^ (544 nm) and Eu^3+^ (616 nm) in the La_0.92_SiBO_5_:0.05Tb^3+^, 0.03Eu^3+^ phosphor at 150 °C have decreased to 82.97% and 84.12% of the initial intensities at 25 °C, respectively. The characteristic emission intensities of Tb^3+^ (544 nm) and Eu^3+^ (616 nm) in the La_0.92_SiBO_5_:0.05Tb^3+^, 0.03Eu^3+^ phosphor at 250 °C have decreased to 77.18% and 67.72% of the initial intensities at 25 °C, respectively. Therefore, this phosphor has good thermal stability for LED applications. The quantum efficiency (QE) is also a critical performance parameter for phosphors used in LEDs. The thermal stability and QE value of phosphor La_0.92_SiBO_5_:0.05Tb^3+^, 0.03Eu^3+^ are better than those of Ca_3_Gd(AlO)_3_(BO_3_)_4_:Tb^3+^, Eu^3+^ [21] and Sr_3_Sc(PO_4_)_3_:Tb^3+^, Eu^3+^ [29].

**Fig 9:**
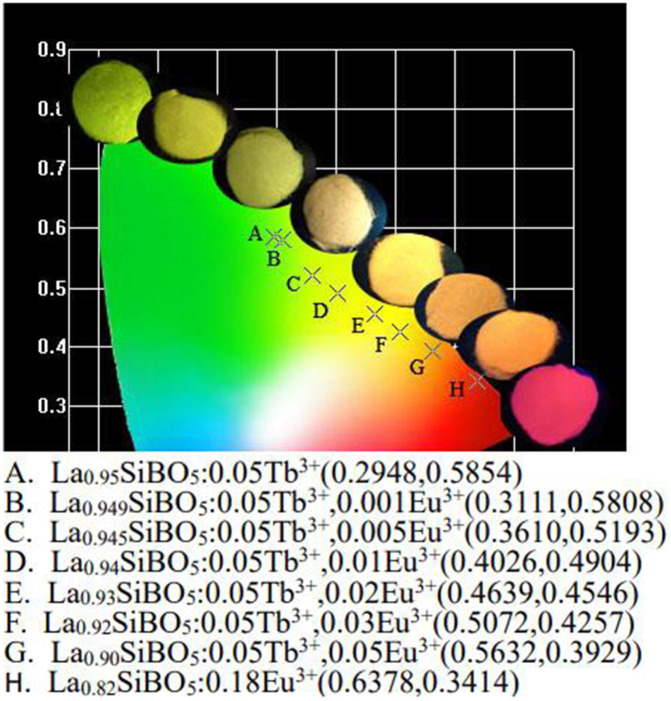
CIE diagram and luminous photos of La_0.95-y_SiBO_5_: 0.05Tb^3+^, yEu^3+^ phosphors.

Fig 9 shows the CIE chromaticity diagram of La_1-x-y_SiBO_5:_x0.05Tb^3+^, yEu^3+^ drawn with CIE 1931 software (E_x_ = 377 nm). The chromaticity coordinates are given in the inset table. The chromaticity coordinates can be changed from green A (0.2948, 0.5854) to yellow E (0.4639, 0.4546) and then to red H (0.6378, 0.3414) by controlling the concentration of Tb^3+^/Eu^3+^. Therefore, the samples have potential applications in multicolor displays and W-LEDs.

## 4. Conclusions

A series of LaSiBO_5_:Tb^3^+, LaSiBO_5_:Eu^3^+ and LaSiBO_5_:Tb^3^+, Eu^3^+ phosphors was synthesized through high-temperature solid-state reaction. XRD results show that the phosphors have pure-phase crystal structures and uniform particle sizes suitable for LED packaging. Among the phosphors, the La_0.93_SiBO_5_:0.05Tb^3^+, 0.02Eu^3^+ phosphor has the strongest luminous intensity. The red and green emission intensity ratio of the La_1-x-y_SiBO_5_:xTb^3^+, yEu^3^+ phosphors decreases with the increase in Eu^3^+ concentration. Therefore, color tone can be effectively adjusted by controlling the doping concentrations of Eu^3^+ and Tb^3^+ ions. When the concentration of Tb^3^+ is 0.05 and that of Eu^3^+ increases from 0.005 to 0.05, the emission intensity of Tb^3^+ decreases gradually, whereas that of Eu^3^+ increases gradually. In addition, color changes from green to yellow and then to red. The results show that the LaSiBO_5_:Tb^3^+, Eu^3^+ phosphors exhibit effective adjustable emission and NUV excitation and can be used as fluorescence conversion materials for display and LED applications.

## Supporting information

S1 Data Minimal raw data collection. (XLS)
